# Coronary Artery Disease and Preoperative Coronary Angiography in Elective Thoracic Endovascular Aortic Repair: A Retrospective Cohort Study

**DOI:** 10.3390/jcdd13060258

**Published:** 2026-06-10

**Authors:** Marwan Hamiko, Lamis Keswani, Ali Bayram, Teresa Rondorf, Andre Spaeth, Miriam Silaschi, Sebastian Zimmer, Chris Probst, Georg Nickenig, Ali El-Sayed Ahmad, Farhad Bakhtiary, Nadjib Schahab

**Affiliations:** 1 Heart Center, Department of Cardiac Surgery, University Hospital Bonn, Venusberg-Campus 1, 53127 Bonn, Germany; 2 Heart Center, Department of Medicine II (Cardiology and Angiology), University Hospital Bonn, Venusberg-Campus 1, 53127 Bonn, Germany; 3 Department of Cardiovascular Surgery, Johannes-Gutenberg University, 55128 Mainz, Germany

**Keywords:** thoracic endovascular aortic repair, coronary artery disease, coronary angiography

## Abstract

(1) Background: Coronary artery disease (CAD) frequently coexists with thoracic aortic disease and may increase the risk of adverse outcomes after thoracic endovascular aortic repair (TEVAR). Whether routine preoperative coronary angiography (CAG) improves outcomes remains unclear. (2) Methods: We retrospectively analyzed 177 patients undergoing elective TEVAR between 2015 and 2025 with a median follow-up of 4.9 years. Two analyses were performed: patients who underwent preoperative CAG versus those who did not, and patients with versus without CAD. Survival was assessed using Kaplan–Meier analysis and overlap-weighted Cox regression. (3) Results: Preoperative CAG was performed in 94 patients (53.1%) and identified newly diagnosed or progressive CAD in 42 (44.7%). Overall, 24 patients (13.6%) underwent coronary revascularization before TEVAR. Patients with CAD were older and had a greater comorbidity burden. Despite these differences, preoperative CAG was not associated with differences in in-hospital mortality (2.1% vs. 6.0%, *p* = 0.159), major adverse cardiovascular events (11.3% vs. 9.0%, *p* = 0.754), or long-term survival (log-rank *p* = 0.10). Patients with CAD showed higher unadjusted long-term mortality than those without CAD (31.7% vs. 17.5%; log-rank *p* = 0.003). However, after overlap weighting, CAD was no longer significantly associated with mortality (adjusted HR 1.4, 95% CI 0.71–2.8). Among patients with angiographically verified coronary disease, preoperative revascularization before TEVAR was not associated with improved long-term survival (HR 2.20, 95% CI 0.69–6.98). (4) Conclusions: Preoperative CAG detects clinically relevant, often unrecognized CAD in a substantial proportion of TEVAR candidates and enables revascularization before surgery. Despite a higher coronary burden, patients who underwent CAG had outcomes comparable to those who did not, and the crude long-term survival disadvantage of CAD was largely explained by the accompanying systemic atherosclerotic burden. Routine preoperative coronary assessment appears justified in elective TEVAR.

## 1. Introduction

Different aortic pathologies are strongly associated with atherosclerosis and, therefore, with the prevalence of cardiovascular disease [1]. However, the relationship between cardiovascular diseases and aortic pathologies remains unclear. Assessment of the cardiovascular status is not routinely performed as a standard preoperative procedure before endovascular repair of aortic pathologies. Previous clinical studies have shown that the prevalence of concomitant coronary artery disease (CAD) in patients with aortic pathologies varies widely, ranging from 5% to 46% [2,3,4]. Notably, several studies have demonstrated that patients with abdominal aortic aneurysms (AAAs) exhibit a higher incidence of CAD, which has been associated with adverse perioperative outcomes, particularly an increased risk of myocardial infarction (MI) and mortality during aortic interventions [5]. In a prior study conducted at the Cleveland Clinic involving 1135 patients who underwent open surgical repair of AAAs, preoperative functional myocardial imaging revealed active myocardial ischemia in 16% of cases. Additionally, among those who underwent coronary angiography (CAG), 29% were found to have severe CAD, needing revascularization [6]. Previous studies have reported a relatively high prevalence of CAD among patients with type B aortic dissection (TBAD) [7,8,9]. Recently, coronary computed tomography (CT) has gained widespread use due to its noninvasive nature and ease of application [10]. Nevertheless, CAG remains a clinically valuable and reliable diagnostic tool, particularly in cases where coronary CT is limited by extensive coronary artery calcification, which may obscure accurate assessment of coronary lesions. Furthermore, CAG proved to be a safe and reliable modality for the detection of concomitant CAD in this specific patient cohort prior to thoracic endovascular aortic repair (TEVAR) [7,8,9]. However, current evidence remains limited regarding the prognostic implications of CAD in patients with thoracic aortic pathologies, particularly in the context of TEVAR.

Therefore, in this actual study we aimed to assess the impact of systematic preoperative coronary assessment and coronary optimization before TEVAR in patients with different aortic pathologies.

## 2. Materials and Methods

### 2.1. Patient Population

**Figure 1:**
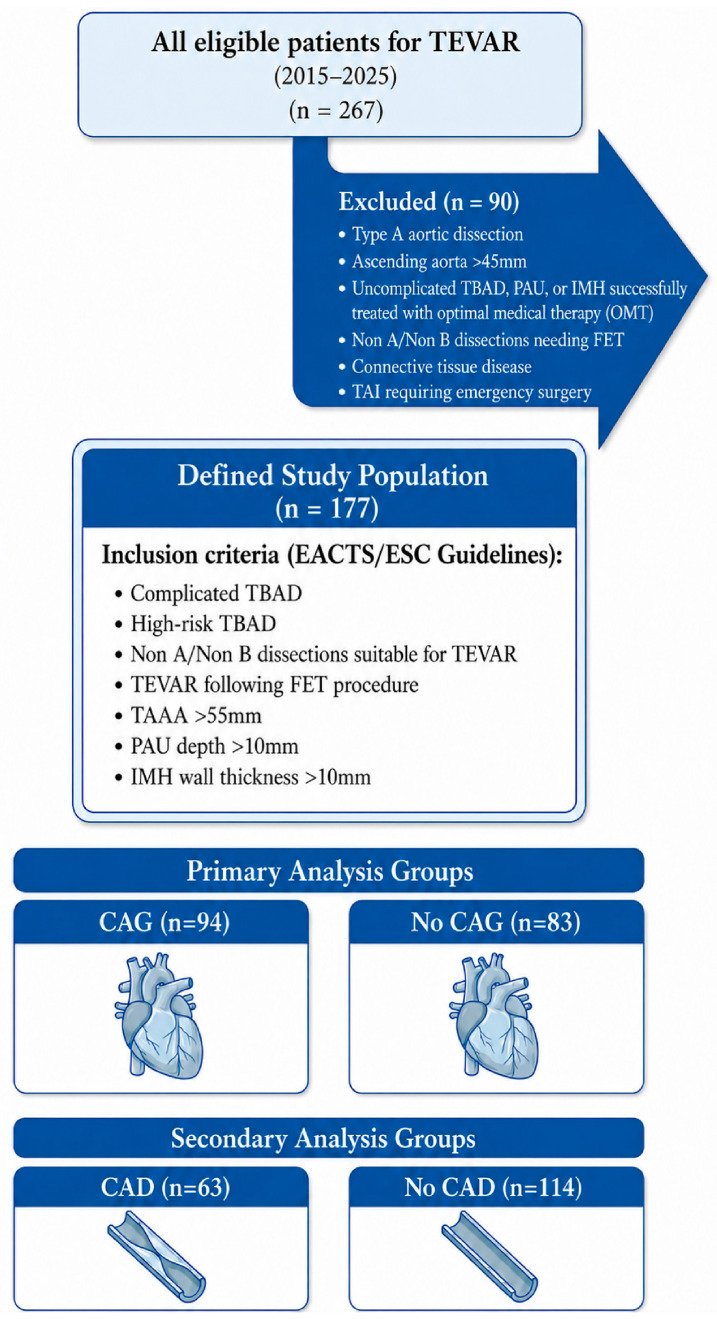
A flow chart demonstrating the inclusion and exclusion criteria for patients in this study. CAD: coronary artery disease; CAG: coronary angiography; FET: frozen elephant trunk; IMH: intramural hematoma; PAU: penetrating aortic ulcer; TAAA: thoracic or thoracoabdominal aortic aneurysm; TAI: traumatic aortic injury; TEVAR: thoracic endovascular aortic repair; TBAD: type B aortic dissection.

We retrospectively analyzed data from patients scheduled for an elective TEVAR procedure from January 2015 to May 2025 at the University Hospital Bonn (Germany). The study hypothesis was that preoperative CAG would frequently identify clinically relevant and potentially treatable CAD and that preoperative coronary optimization might attenuate the expected perioperative risk associated with concomitant CAD. Accordingly, patients were included in this cohort irrespective of the underlying thoracic aortic pathology. Therefore, all patients with complicated TBAD, TBAD with high-risk features for the development of complicated dissection, Non A/Non B aortic dissection suitable for TEVAR, a prior frozen elephant trunk (FET) procedure requiring TEVAR extension for treatment completion, thoracic or thoracoabdominal aortic aneurysm (TAAA), penetrating aortic ulcer (PAU), or intramural hematoma (IMH) indicated for TEVAR were included in this actual study (Figure 1).

The exclusion criteria were patients with type A aortic dissection, aneurysm of the ascending aorta >45 mm, or uncomplicated TBAD, PAU or IMH treated with optimal medical therapy; patients with connective tissue disease; patients with traumatic aortic injury (TAI) needing emergency TEVAR; and patients with Non A/Non B dissection scheduled for an FET procedure. Indication for surgery was in accordance with the former and current EACTS/ESC guidelines. [11,12] Figure 1 presents a flowchart outlining the inclusion and exclusion criteria for patients analyzed in the present study.

Baseline clinical characteristics, including patient demographics, medical history, presenting clinical features, imaging findings, and postoperative outcome, were obtained through a retrospective review of medical records.

The collection of pre- and postoperative pharmacological data, including statin therapy, antiplatelet regimens, beta-blocker use, and secondary cardiovascular prevention, was not a prespecified endpoint of this retrospective study. Consequently, reliable and systematic patient-level medication data could not be consistently reconstructed for all individuals across the full ten-year study period and were therefore not included in the formal statistical analysis. To provide clinical context, institutional postoperative pharmacological management of TEVAR patients during the study period is summarized in the Supplementary Methods (Supplementary File S1).

### 2.2. Procedural Details and Strategies

TEVAR was performed in a hybrid operating room for a broad spectrum of pathologies of the thoracic descending aorta after review of the CT scans and sizing of the aortic diameter, as well as proximal and landing zones. Endovascular stent grafts, selected with 5–15% oversizing relative to the native aortic diameter, were delivered via transfemoral access under general anesthesia. In patients with proximal landing zones involving arch segments (zones 1 or 2) or in those with anatomical variants such as an aberrant right subclavian artery, adjunctive hybrid procedures, including left carotid–subclavian bypass (CSB), were performed as needed, following a detailed evaluation of the individual aortic anatomy and patient preference. In few cases right and left CSB were necessary. The decision to proceed with TEVAR was based on a multidisciplinary consensus, and TEVAR was performed only after fully informed consent was obtained from the patient and their family.

### 2.3. Preoperative Diagnostic Assessment and Management of CAD

All patients presenting to our institution with pathologies of the thoracic descending aorta underwent a comprehensive preoperative evaluation, provided the intervention was not performed on an emergency basis. As part of the standard diagnostic work-up, a contrast-enhanced CT scan was performed in all cases and served as the cornerstone for procedural planning. Additionally, each patient received a 12-lead electrocardiogram (ECG), pulmonary function testing, duplex ultrasonography of the supra-aortic vessels, and transthoracic echocardiography.

If prior CAG was available, it was considered valid only if performed within the preceding 12 months. Before 2022, invasive CAG was not routinely included in the institutional preoperative diagnostic work-up for elective TEVAR candidates. Consequently, patients treated before implementation of the routine CAG protocol generally did not undergo systematic invasive coronary assessment unless it was clinically indicated or previously performed. In these patients, the presence of CAD was determined based on documented medical history, referral letters, prior coronary angiography findings, and available cardiovascular records at the time of presentation. Following two intraoperative incidents of acute MI during anesthesia induction in patients with previously unrecognized CAD, routine invasive CAG was adopted in 2022 as a standard component of the preoperative work-up for TEVAR procedures in patients older than 40 years.

CAG was mostly performed via right radial artery access. CAD was defined as the presence of significant stenosis, characterized by a ≥50% reduction in luminal diameter in a major epicardial coronary artery. Based on the number of affected vessels, patients with CAD were further classified as having single-vessel or multi-vessel disease. CAG findings were independently reviewed and interpreted by two experienced cardiologists.

In cases of significant coronary artery stenosis, percutaneous coronary intervention (PCI) with stent implantation was typically performed prior to the planned TEVAR, provided there was no indication for coronary artery bypass grafting (CABG). Following PCI, all patients received guideline-recommended dual antiplatelet therapy (DAPT). TEVAR was then electively scheduled 4 to 6 weeks after the coronary intervention. Patients who required CABG and had an indication for TEVAR underwent CABG first, followed by TEVAR in a staged approach. Patients who received simultaneous CABG and TEVAR were excluded from this actual study.

### 2.4. Primary and Secondary Endpoints

The primary endpoints of this study were 30-day and in-hospital mortality. The secondary endpoint was a composite outcome comprising cardiovascular deaths and cardiovascular events (major adverse clinical events (MACEs)). MACEs were defined as the occurrence of any of the following: MI, stroke, retrograde type A aortic dissection, limb ischemia, visceral ischemia or spinal cord ischemia. Data on survival status were collected through structured telephone interviews or during scheduled outpatient follow-up visits at predefined intervals.

### 2.5. Study Groups

For the primary analysis, patients were stratified according to the performance of preoperative CAG, reflecting the institutional diagnostic strategy during the study period. Patients who underwent preoperative CAG comprised the CAG group (*n* = 94), whereas patients managed without routine invasive coronary assessment comprised the no-CAG group (*n* = 83). This grouping enabled comparison between patients undergoing systematic invasive coronary assessment and those managed without routine CAG before elective TEVAR.

For the secondary analysis, patients were additionally categorized according to the presence or absence of CAD (CAD group, *n* = 63; no-CAD group, *n* = 114). CAD status was determined by preoperative CAG when available or by documented medical history, prior coronary investigations, and referral records in patients treated before the implementation of routine CAG.

### 2.6. Statistical Analysis

Continuous variables are presented as the median and interquartile range (IQR) and compared between groups using the Mann–Whitney U test; categorical variables are expressed as frequencies and percentages and compared using Fisher’s exact test. To quantify between-group imbalances in baseline characteristics, standardized mean differences (SMDs) were computed for each variable, with an absolute SMD < 0.10 prespecified as the threshold for adequate balance.

Two complementary comparisons were prespecified. The first compared patients who underwent preoperative CAG with those who did not, in the entire cohort without adjustment, since this comparison corresponds to the clinical strategy under evaluation. The diagnostic and therapeutic yield of CAG (newly diagnosed or progressive coronary artery disease [CAD] and coronary revascularization performed before TEVAR) was summarized descriptively within the CAG group.

The second comparison evaluated the prognostic impact of CAD (CAD vs. no CAD). As the covariates that define the coronary risk profile (age, sex, hypertension, diabetes mellitus, hyperlipidemia, chronic kidney disease, peripheral arterial disease, cerebral arterial disease and current smoking) are themselves determinants of CAD, the CAD and no-CAD groups lacked sufficient common support for one-to-one propensity-score matching (PSM) to achieve covariate balance. Adjustment was therefore performed by propensity-score overlap weighting, in which the propensity score was estimated by logistic regression on the nine covariates above, and each patient was weighted by the probability of belonging to the opposite group. Overlap weighting targets the population with clinical equipoise and yields exact balance of the means of all included covariates by construction. Balance was assessed by SMDs before and after weighting. Weighted event rates were compared using design-based (Rao–Scott) tests, and weighted survival was compared using a Cox model with overlap weights and robust (sandwich) standard errors. The CAD analysis was repeated within the subgroup of patients with angiographically verified coronary status (i.e., those who underwent CAG) as a sensitivity analysis.

Long-term survival was estimated by the Kaplan–Meier method and compared between groups by the log-rank test (unadjusted) and by the overlap-weighted Cox model (adjusted). All tests were two-sided, and a *p*-value < 0.05 was considered statistically significant. Analyses were performed using R (R Foundation for Statistical Computing, version 4.6.0, Vienna, Austria) with the WeightIt (version 1.7.0), cobalt (version 4.6.2), survey (version 4.5), survival (version 3.8-6), survminer (version 0.5.2), smd (version 0.8.0), gtsummary (version 2.5.0) and tidyverse packages (version 2.0.0).

## 3. Results

### 3.1. Patient Population

A total of 177 patients underwent TEVAR during the study period; 94 (53.1%) underwent preoperative CAG and 83 (46.9%) did not. CAD was present in 63 patients (35.6%) and absent in 114 (64.4%). Forty deaths were recorded over a mean follow-up of 41.8 months (median 2.9 years). Patients in the CAG group were older (median 72.0 [IQR 60.2–77.0] vs. 62.0 [55.0–73.0] years; SMD 0.432) and had a higher prevalence of hyperlipidemia (36.2% vs. 22.9%; SMD 0.294), current smoking (39.4% vs. 25.3%; SMD 0.304), prior stroke (14.9% vs. 4.8%; SMD 0.343) and atrial fibrillation (19.1% vs. 10.8%; SMD 0.234); sex, body mass index (BMI), hypertension, diabetes mellitus, baseline renal function, COPD, peripheral (pAVD) and cerebral arterial vascular disease (cAVD) and bovine arch anatomy were comparable between the groups (Table 1).

### 3.2. Coronary Status and Diagnostic Findings of Coronary Angiography

The coronary profile is summarized in Table 2. Pre-existing CAD before TEVAR was documented in 51 patients (28.8%); a history of MI was recorded in nine patients (5.1%) and prior PCI or coronary stenting in 14 patients (7.9%). The prevalence of CAD was substantially higher among patients who underwent CAG than among those who did not (39.4% vs. 16.9%, *p* = 0.001). Among the 94 patients who underwent preoperative CAG, newly diagnosed or progressive CAD was identified in 42 patients (44.7% of the CAG group; 23.7% of the entire cohort), and 24 patients (25.5% of the CAG group; 13.6% of the entire cohort) underwent coronary revascularization (PCI or CABG) before TEVAR (Table 2).

### 3.3. Procedural Details

Detailed procedural characteristics are presented in Supplementary Table S1. Procedural characteristics were largely comparable between the CAG and no-CAG groups (Table S1). Coverage of the left subclavian artery (LSA; 36.6% vs. 44.6%; *p* = 0.287) and carotid–subclavian bypass (28.3% vs. 39.8%; *p* = 0.113) did not differ significantly, whereas plug occlusion of the LSA was performed less frequently in the CAG group (5.1% vs. 18.8%; *p* = 0.031).

### 3.4. Postoperative Outcomes According to Preoperative CAG Status (Full Cohort)

**Figure 2:**
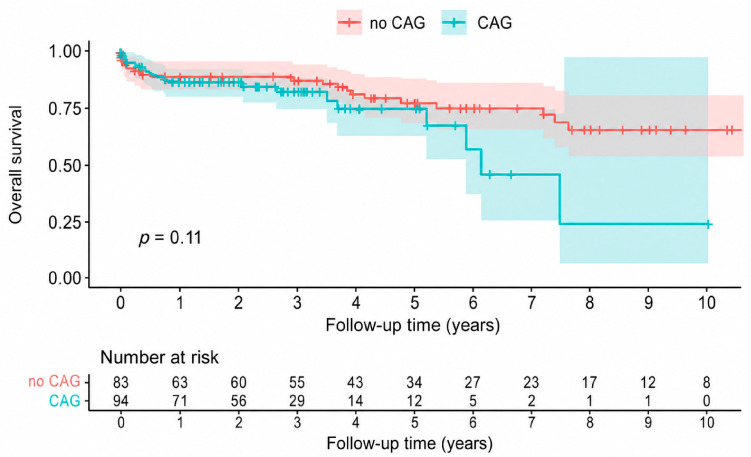
Kaplan–Meier curve for cumulative survival rates and long-term mortality. CAG: coronary angiography.

Postoperative outcomes in the unadjusted full cohort are shown in Table 3. None of the prespecified perioperative outcomes differed significantly between the CAG and no-CAG groups. In-hospital mortality was 2.1% in the CAG group vs. 6.0% in the no-CAG group (*p* = 0.255), and 30-day mortality was 4.3% vs. 4.8% (*p* > 0.999). MACEs occurred in 14 patients (14.9%) in the CAG group and 11 (13.3%) in the no-CAG group (*p* = 0.754). During follow-up, mortality was 21.3% (20/94) in the CAG group and 24.1% (20/83) in the no-CAG group, and Kaplan–Meier survival did not differ between the groups (log-rank *p* = 0.11; Figure 2).

### 3.5. Prognostic Impact of Coronary Artery Disease: Overlap-Weighted Analysis

Before adjustment, CAD and no-CAD patients differed markedly across the coronary risk profile, with a maximum absolute SMD of 0.87 (Table 4). One-to-one PSM could not balance these covariates because of limited common support; propensity-score overlap weighting achieved exact balance, reducing all covariate SMDs to ≤0.001 (Table S2; Figure S1), with an effective sample size of 47 CAD and 67 no-CAD patients. In the overlap-weighted cohort, postoperative outcomes did not differ significantly between CAD and no-CAD patients (Table 5).

**Figure 3:**
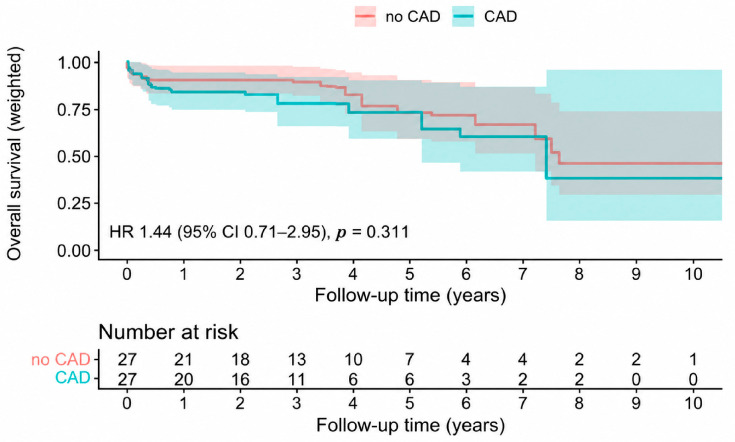
Kaplan–Meier curve for overlap-weighted survival in CAD and no-CAD patients. CAD: coronary artery disease; CI: confidence interval; HR: hazard ratio.

Unadjusted long-term survival was significantly lower in patients with CAD than in those without (follow-up mortality 31.7% [20/63] vs. 17.5% [20/114]; log-rank *p* = 0.003; unadjusted hazard ratio [HR] 2.48; Figure S2). After overlap weighting, the difference was attenuated and no longer statistically significant (weighted HR 1.44, 95% CI 0.71–2.95; *p* = 0.311; Figure 3).

**Figure 4:**
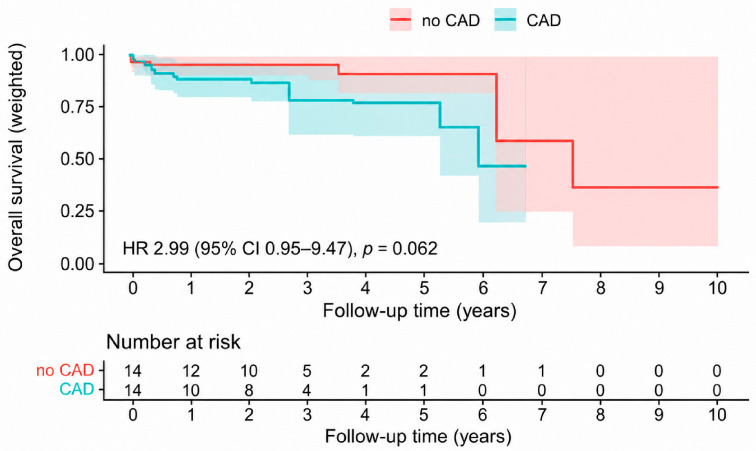
Kaplan–Meier curve for sensitivity analysis in CAD within CAG patients (verified coronary status). CAD: coronary artery disease; CAG: coronary angiography; CI: confidence interval; HR: hazard ratio.

In the sensitivity analysis restricted to patients with angiographically verified coronary status (CAG group, *n* = 94), the overlap-weighted association between CAD and mortality was greater and of borderline significance (weighted HR 2.99, 95% CI 0.95–9.47; *p* = 0.062, Figure 4).

## 4. Discussion

The role of systematic preoperative coronary assessment in patients scheduled for TEVAR remains poorly defined. Current guidelines for the management of aortic disease and for perioperative cardiovascular evaluation give no clear recommendation on routine invasive coronary diagnostics before elective TEVAR, regardless of the underlying aortic pathology [11,12,13]. This lack of guidance is notable given that atherosclerosis represents a key pathophysiological substrate for both aortic disease and CAD, and that patients undergoing TEVAR frequently exhibit a high burden of systemic cardiovascular comorbidities [14,15,16].

At our institution, invasive CAG was added to the standardized preoperative work-up in 2022, after two ischemic events during anesthesia induction exposed the vulnerability of TEVAR candidates with unrecognized CAD. This protocol change created a quasi-before–after structure within our cohort: patients treated before 2022 underwent CAG only selectively, based on clinical suspicion and referral documentation, while those treated from 2022 onward were assessed systematically. This heterogeneous diagnostic exposure across the study period introduces a form of temporal bias that must be considered when interpreting outcome comparisons. Secular trends in patient selection, anesthetic management, and perioperative care across a decade-long observation period cannot be fully accounted for, and this limitation is acknowledged explicitly below. With these caveats in mind, the present analysis is best understood as an observational, hypothesis-generating evaluation of the diagnostic yield and potential clinical relevance of routine preoperative CAG in elective TEVAR candidates, not as a demonstration of causal benefit.

In the present cohort, pre-existing CAD was documented in 28.8% of patients, in line with previous reports on thoracic aortic disease and close to the 26.5% prevalence Li et al. described in type B aortic dissection [9]. Among patients who underwent CAG, invasive assessment uncovered newly diagnosed or progressive CAD in 44.7% (23.7% of the whole cohort) and led to revascularization before TEVAR in a quarter of the CAG group (13.6% of the total cohort). The prevalence of CAD was three times higher in patients who underwent CAG than in those who did not (52.1% vs. 16.9%), reflecting both the higher cardiovascular risk profile that prompted referral for CAG and the additional disease that invasive assessment itself revealed. Clinically relevant CAD is therefore common and frequently unrecognized in TEVAR candidates, and symptom-based or noninvasive screening is likely to miss a substantial proportion of it. Whether systematic identification and treatment of these lesions translates into improved outcomes, however, cannot be established from the present data alone and requires prospective evaluation.

Despite carrying a markedly higher coronary burden, patients who underwent CAG did not have worse perioperative or long-term outcomes than those who did not. In-hospital mortality (2.1% vs. 6.0%), 30-day mortality (4.3% vs. 4.8%), MACEs (14.9% vs. 13.3%), and follow-up survival (log-rank *p* = 0.11) were comparable between the two groups. While consistent with a detect-and-treat effect whereby preoperative identification and revascularization of significant coronary lesions may attenuate the elevated baseline risk of the CAG group, these findings remain associative in nature and do not permit causal inference. The absence of a contemporaneous, prospectively defined control group and the temporal heterogeneity of the cohort preclude causal conclusions. Alternative explanations, including differences in patient selection, institutional learning effects, and improvements in anesthetic and perioperative management over the study period, cannot be excluded as contributors to the observed outcome pattern.

The contrast with Li et al. is instructive in this context, though it must be interpreted with caution [9]. In their dissection cohort, CAG was also performed before TEVAR, but revascularization was deferred until afterward, and CAD was independently associated with worse short-term outcomes. In the present cohort, by contrast, significant coronary artery stenoses were treated before TEVAR, and early outcomes did not differ by CAD status. This divergence is hypothesis-generating: the timing of revascularization, rather than the presence of CAD alone, may matter for perioperative prognosis, possibly by reducing the risk of myocardial ischemia during periods of hemodynamic stress such as anesthesia induction, rapid ventricular pacing, or transient hypotension at stent-graft deployment [17,18]. However, the two studies differ substantially in their patient selection criteria, aortic pathology spectrum, institutional practice, and era of treatment, and these differences may fully or partially account for the divergent findings. The contrast between the two cohorts therefore generates a testable hypothesis but does not establish a causal hierarchy between revascularization strategies.

The prognostic weight of CAD itself was examined in the weighted analysis, where its interpretation requires particular care considering the substantial comorbidity burden that accompanies CAD in this population. Crude long-term survival was significantly lower in patients with CAD (HR 2.48; log-rank *p* = 0.003), but after overlap weighting balanced the coronary risk profile across the groups, the association was markedly attenuated and lost its statistical significance (weighted HR 1.44, 95% CI 0.71–2.95). This pattern is consistent with the interpretation that much of the apparent survival disadvantage attributed to CAD is in fact carried by the wider systemic atherosclerotic burden that accompanies it, encompassing advanced age, diabetes mellitus, hyperlipidemia, chronic kidney disease, peripheral arterial disease, and cerebral arterial disease, rather than by CAD per se [19,20,21,22,23,24,25,26]. Viewed from this perspective, CAD behaves primarily as a marker of global cardiovascular risk in this population, rather than as an isolated, independently modifiable driver of late mortality. Alternative explanations for the attenuation of the weighted association, including the relatively small effective sample size after weighting and residual confounding from unmeasured variables such as pharmacological secondary prevention, cannot be excluded. The borderline weighted association observed in the subgroup with angiographically verified coronary status (weighted HR 2.99, 95% CI 0.95–9.47) indicates that a residual, CAD-specific contribution to long-term risk cannot be ruled out, and this is the subgroup least susceptible to misclassification bias. Whether this signal reflects a true independent CAD effect or continued confounding by unmeasured risk factors warrants investigation in larger, prospectively designed studies.

From a clinical perspective, the present findings are consistent with, but do not prove, a role for routine invasive coronary assessment in selected patients undergoing elective TEVAR. CAG demonstrated a high diagnostic yield and direct therapeutic consequences without procedure-related aortic complications; radial or brachial access avoids manipulation of the diseased aorta and integrates efficiently into the standard angiographic workflow [18,27]. At the same time, the attenuation of the CAD–mortality association after adjustment for the broader vascular comorbidity profile underscores that preoperative CAG cannot be expected to remove the long-term mortality risk associated with advanced systemic atherosclerosis. Risk stratification in TEVAR candidates should therefore extend beyond coronary anatomy to encompass the full burden of vascular comorbidity, including cerebrovascular disease, peripheral arterial disease, renal function, and metabolic risk factors, as these are the determinants of long-term prognosis in this population. Ultimately, whether a strategy of systematic preoperative coronary assessment reduces perioperative ischemic events and improves long-term survival in elective TEVAR candidates can only be established by prospective, randomized trials. The present observational data provide a hypothesis-generating foundation and a rationale for such an investigation.

## 5. Conclusions

In patients undergoing elective TEVAR, CAD is common and frequently undiagnosed without systematic preoperative assessment. Routine invasive CAG identified clinically relevant CAD in a substantial proportion of patients and enabled preemptive revascularization in a meaningful subset. Despite carrying a higher coronary burden, patients who underwent CAG had outcomes comparable to those for patients who did not, a finding consistent with, but not proof of, a detect-and-treat effect. The long-term survival disadvantage associated with CAD was largely attenuated after adjustment for the accompanying systemic atherosclerotic burden, suggesting that CAD functions primarily as a marker of global cardiovascular risk rather than as an independent determinant of late mortality in this population. These findings are observational and hypothesis-generating; they should not be interpreted as establishing a causal benefit of routine preoperative coronary angiography. Prospective randomized trials are needed to determine whether systematic preoperative coronary assessment reduces perioperative ischemic events and improves long-term survival, and to provide the evidence base required for guideline-level recommendations in elective TEVAR candidates.

## 6. Limitations

This study has several limitations. First, its retrospective, single-center design carries selection bias and limits causal inference; in particular, the decision to perform CAG was clinical rather than randomized. Second, routine invasive CAG was implemented only after 2022, introducing temporal bias and a quasi-before–after study structure. In patients treated before the implementation of routine CAG, CAD status was based on documented medical history, referral records, and prior coronary investigations rather than systematic invasive coronary assessment, introducing a potential risk of misclassification. We addressed this with a sensitivity analysis confined to angiographically assessed patients. Overlap weighting balances measure covariates but cannot account for unmeasured confounding, and the modest number of events limits the precision of the survival estimates. Third, the cohort included heterogeneous aortic pathologies, though atherosclerosis contributes to all of them and is shared with CAD. Finally, differences in post-TEVAR medical therapy and secondary prevention were not available throughout follow-up; therefore, they could not be analyzed and may have influenced long-term outcomes.

## Figures and Tables

**Table 1:** Patient characteristics by preoperative CAG.

	Overall (*n* = 177)	No CAG (*n* = 83)	CAG (*n* = 94)	SMD
Age (years), median (IQR)	68.0 (58.0–76.0)	62.0 (55.0–73.0)	72.0 (60.0–77.0)	0.432
Male sex, *n* (%)	114 (64.4%)	56 (67.5%)	58 (61.7%)	0.121
BMI (kg/m^2^), median (IQR)	25.8 (23.8–30.0)	24.9 (23.8–29.3)	27.1 (23.7–30.2)	0.083
Hypertension, *n* (%)	153 (86.4%)	72 (86.7%)	81 (86.2%)	0.017
Hyperlipidemia, *n* (%)	53 (29.9%)	19 (22.9%)	34 (36.2%)	0.294
Diabetes mellitus, *n* (%)	23 (13.0%)	10 (12.0%)	13 (13.8%)	0.053
CKD, *n* (%)	36 (20.3%)	15 (18.1%)	21 (22.3%)	0.106
Baseline creatinine (mg/dL), median (IQR)	0.9 (0.8–1.1)	0.9 (0.8–1.2)	0.9 (0.8–1.1)	0.000
Current smoker, *n* (%)	58 (32.8%)	21 (25.3%)	37 (39.4%)	0.304
COPD, *n* (%)	30 (16.9%)	11 (13.3%)	19 (20.2%)	0.187
pAVD, *n* (%)	27 (15.3%)	11 (13.3%)	16 (17.0%)	0.105
cAVD, *n* (%)	20 (11.3%)	9 (10.8%)	11 (11.7%)	0.027
Prior stroke, *n* (%)	18 (10.2%)	4 (4.8%)	14 (14.9%)	0.343
Atrial fibrillation, *n* (%)	27 (15.3%)	9 (10.8%)	18 (19.1%)	0.234
Bovine arch, *n* (%)	15 (8.5%)	5 (6.0%)	10 (10.6%)	0.168

BMI: body mass index; CAG: coronary angiography; cAVD: cerebral arterial vascular disease; CKD: chronic kidney disease; COPD: chronic obstructive pulmonary disease; IQR: interquartile range; pAVD: peripheral arterial vascular disease; SMD: standardized mean difference.

**Table 2:** Coronary artery disease status and coronary angiography findings.

	Overall (*n* = 177)	No CAG (*n* = 83)	CAG (*n* = 94)	*p*-Value
Prior MI, *n* (%)	9 (5.1%)	0 (0.0%)	9 (9.6%)	0.004
Pre-TEVAR known CAD, *n* (%)	51 (28.8%)	14 (16.9%)	37 (39.4%)	0.001
Prior PCI/stenting, *n* (%)	15 (8.5%)	2 (2.4%)	13 (13.8%)	0.007
De novo/progressive CAD, *n* (%)	42 (23.7%)	0 (0.0%)	42 (44.7%)	<0.001
Revascularization before TEVAR, *n* (%)	24 (13.5%)	0 (0.0%)	24 (25.5%)	<0.001

CAD: coronary artery disease; CAG: coronary angiography; MI: myocardial infarction; PCI: percutaneous coronary intervention; TEVAR: thoracic endovascular aortic repair.

**Table 3:** Postoperative outcomes stratified by CAG status (full cohort).

	Total (*n* = 177)	No CAG (*n* = 83)	CAG (*n* = 94)	*p*-Value
Rupture, *n* (%)	1 (0.6%)	1 (1.2%)	0 (0.0%)	0.469
Endoleak, *n* (%)	26 (14.7%)	14 (16.9%)	12 (12.8%)	0.578
Conversion to open surgery, *n* (%)	2 (1.1%)	0 (0.0%)	2 (2.2%)	0.499
Graft migration, *n* (%)	2 (1.1%)	2 (2.4%)	0 (0.0%)	0.218
Graft infection, *n* (%)	1 (0.6%)	1 (1.2%)	0 (0.0%)	0.469
Pneumonia, *n* (%)	16 (9.0%)	9 (10.8%)	7 (7.4%)	0.445
Tracheostomy, *n* (%)	7 (4.0%)	5 (6.0%)	2 (2.1%)	0.255
Retrograde type A dissection, *n* (%)	4 (2.3%)	2 (2.4%)	2 (2.1%)	>0.999
Inguinal wound infection, *n* (%)	10 (5.6%)	5 (6.0%)	5 (5.3%)	>0.999
MACE, *n* (%)	25 (14.1%)	11 (13.3%)	14 (14.9%)	0.754
Stroke, *n* (%)	5 (2.8%)	3 (3.6%)	2 (2.1%)	0.666
MI, *n* (%)	3 (1.7%)	1 (1.2%)	2 (2.1%)	>0.999
Spinal cord ischemia, *n* (%)	8 (4.5%)	4 (4.8%)	4 (4.3%)	>0.999
Delirium, *n* (%)	15 (8.5%)	10 (12.0%)	5 (5.3%)	0.175
Bowel ischemia, *n* (%)	3 (1.7%)	2 (2.4%)	1 (1.1%)	0.601
Vascular injury, *n* (%)	12 (6.8%)	3 (3.6%)	9 (9.6%)	0.142
Limb ischemia, *n* (%)	10 (5.6%)	2 (2.4%)	8 (8.5%)	0.106
Acute kidney injury, *n* (%)	20 (11.3%)	12 (14.5%)	8 (8.5%)	0.241
Need of dialysis, *n* (%)	9 (5.1%)	5 (6.0%)	4 (4.3%)	0.736
In-hospital mortality, *n* (%)	7 (4.0%)	5 (6.0%)	2 (2.1%)	0.255
30-day mortality, *n* (%)	8 (4.5%)	4 (4.8%)	4 (4.3%)	>0.999
Re-intervention during FU, *n* (%)	50 (30.9%)	24 (32.4%)	26 (29.5%)	0.853
Further TEVAR during FU, *n* (%)	32 (20.8%)	11 (16.2%)	21 (24.4%)	0.117

CAG: coronary angiography; FU: follow-up; MACE: major adverse cardiovascular event; MI: myocardial infarction; TEVAR: thoracic endovascular aortic repair.

**Table 4:** Patient characteristics by preoperative CAD.

	Overall (*n* = 177)	No CAD (*n* = 114)	CAD (*n* = 63)	SMD
Age (years), median (IQR)	68.0 (58.0–76.0)	61.0 (55.0–73.0)	74.0 (67.0–79.0)	0.821
Male sex, *n* (%)	114 (64.4%)	71 (62.3%)	43 (68.3%)	0.126
BMI (kg/m^2^), median (IQR)	25.8 (23.8–30.0)	25.7 (23.8–30.0)	27.3 (22.7–30.0)	0.001
Hypertension, *n* (%)	153 (86.4%)	101 (88.6%)	52 (82.5%)	0.173
Hyperlipidemia, *n* (%)	53 (29.9%)	23 (20.2%)	30 (47.6%)	0.606
Diabetes mellitus, *n* (%)	23 (13.0%)	9 (7.9%)	14 (22.2%)	0.409
CKD, *n* (%)	36 (20.3%)	17 (14.9%)	19 (30.2%)	0.371
Baseline creatinine (mg/dL), median (IQR)	0.9 (0.8–1.1)	0.9 (0.8–1.1)	1.0 (0.8–1.2)	0.165
Current smoker, *n* (%)	58 (32.8%)	32 (28.1%)	26 (41.3%)	0.280
COPD, *n* (%)	30 (16.9%)	17 (14.9%)	13 (20.6%)	0.150
pAVD, *n* (%)	27 (15.3%)	8 (7.0%)	19 (30.2%)	0.623
cAVD, *n* (%)	20 (11.3%)	9 (7.9%)	11 (17.5%)	0.291
Prior stroke, *n* (%)	18 (10.2%)	6 (5.3%)	12 (19.0%)	0.432
Atrial fibrillation, *n* (%)	27 (15.3%)	14 (12.3%)	13 (20.6%)	0.227
Bovine arch, *n* (%)	15 (8.5%)	9 (7.9%)	6 (9.5%)	0.058

BMI: body mass index; CAD: coronary artery disease; cAVD: cerebral arterial vascular disease; CKD: chronic kidney disease; COPD: chronic obstructive pulmonary disease; IQR: interquartile range; pAVD: peripheral arterial vascular disease.

**Table 5:** Postoperative outcomes according to coronary artery disease status (overlap-weighted analysis).

Outcome	No CAG (wt%))	CAG (wt%)	*p*-Value (Weighted)
Rupture	0.7%	0.0%	0.000
Endoleak	15.2%	18.1%	0.686
Conversion to open surgery	1.4%	0.0%	0.000
Graft migration	0.2%	0.0%	0.000
Graft infection	0.5%	0.0%	0.000
Pneumonia	4.1%	11.5%	0.081
Tracheostomy	3.0%	4.3%	0.686
Retrograde type A dissection	1.7%	3.1%	0.622
Inguinal wound infection	2.3%	11.1%	0.048
MACE	6.3%	14.9%	0.093
Stroke	1.8%	2.6%	0.698
MI	0.0%	1.7%	0.000
Spinal cord ischemia	0.7%	3.7%	0.043
Delirium	12.1%	5.2%	0.210
Bowel ischemia	0.0%	1.6%	0.004
Vascular injury	5.0%	6.6%	0.669
Limb ischemia	2.9%	7.7%	0.216
Acute kidney injury	12.5%	6.9%	0.261
Need of dialysis	1.7%	4.4%	0.237
In-hospital mortality	4.0%	5.4%	0.747
30-day mortality	5.1%	5.6%	0.903
Re-intervention during FU	31.3%	29.6%	0.849
Further TEVAR during FU	20.8%	22.9%	0.798

CAG: coronary angiography; FU: follow-up; MACE: major adverse cardiovascular event; MI: myocardial infarction; TEVAR: thoracic endovascular aortic repair.

## Data Availability

The data underlying this study were obtained retrospectively from clinical records and contain sensitive personal health information. Due to privacy regulations and ethical considerations, the raw data cannot be made publicly available. Researchers with a legitimate interest may request access to anonymized or aggregated data, subject to approval by the responsible ethics committee and in compliance with applicable data protection laws.

## Supplementary Materials

The following supporting information can be downloaded at https://www.mdpi.com/article/10.3390/jcdd13060258/s1, Methods File S1: Institutional postoperative pharmacological management during the study period, Table S1: Procedural details, Table S2: Covariate balance before vs. after overlap weighting, Figure S1: Love plot (overlap weighting). Covariate valance: unweighted vs. overlap weighted, Figure S2: Kaplan–Meier curve for cumulative survival rates and long-term mortality.

## Abbreviations

The following abbreviations are used in this manuscript:

AAA	Abdominal aortic aneurysm
AKI	Acute kidney injury
BMI	Body mass index
CABG	Coronary artery bypass grafting
CAD	Coronary artery disease
CAG	Coronary angiography
cAVD	Cerebral arterial vascular disease
CI	Confidence interval
CKD	Chronic kidney disease
COPD	Chronic obstructive pulmonary disease
CSB	Carotid–subclavian bypass
CT	Computed tomography
DAPT	Dual antiplatelet therapy
ECG	Electrocardiogram
ESC	European Society of Cardiology
EACTS	European Association for Cardio-Thoracic Surgery
FET	Frozen elephant trunk
FU	Follow-up
HR	Hazard ratio
IMH	Intramural hematoma
IQR	Interquartile range
LSA	Left subclavian artery
MACE	Major adverse cardiovascular event
MI	Myocardial infarction
PAU	Penetrating aortic ulcer
PCI	Percutaneous coronary intervention
pAVD	Peripheral arterial vascular disease
PSM	Propensity score matching
SMD	Standardized mean difference
TAI	Traumatic aortic injury
TAAA	Thoracic or thoracoabdominal aortic aneurysm
TBAD	Type B aortic dissection
TEVAR	Thoracic endovascular aortic repair
