# Supplementary material for: Coronary Artery Disease and Preoperative Coronary Angiography in Elective Thoracic Endovascular Aortic Repair: A Retrospective Cohort Study
Source: J Cardiovasc Dev Dis. 2026 Jun 10;13(6):258. doi: 10.3390/jcdd13060258 (PMC13301176; doi:10.3390/jcdd13060258)
Supplement: Supplementary file 1 [file jcdd-13-00258-s001.zip › jcdd-4266504-supplementary.pdf]

Supplementary Files

Supplementary Methods S1. Institutional Postoperative Pharmacological Management During the Study Period

Although structured patient-level medication data were not systematically available for formal statistical analysis, institutional postoperative pharmacological management generally followed standardized practice during the study period.

Patients undergoing TEVAR were routinely discharged on statin therapy, beta-blocker treatment, and at minimum aspirin monotherapy as the pharmacological backbone of secondary cardiovascular prevention and aortic disease management. Antiplatelet therapy was individualized according to coronary artery disease (CAD) status and coronary intervention.

Patients without evidence of CAD generally received aspirin monotherapy for three months after TEVAR. Patients with established CAD routinely received lifelong aspirin therapy. Patients undergoing preoperative percutaneous coronary intervention (PCI), received guideline-recommended dual antiplatelet therapy (DAPT), consisting of aspirin and clopidogrel. Patients undergoing concomitant supra-aortic vessel transposition or bypass procedures typically received one month of DAPT according to institutional practice.

These treatment pathways reflect institutional standards of care during the study period and are provided to contextualize perioperative management; however, individual medication regimens were not systematically captured and therefore could not be included in formal statistical analyses.

Supplementary Table S1

Table S1. Procedural Details.

	Overall (n=177)	No CAG (n=83)	CAG (n=114)	p-value
LSA coverage, n (%)	71 (40.3%)	37 (44.6%)	34 (36.6%)	0.29
CSB; n (%)	59 (33.7%)	33 (39.8%)	26 (28.3%)	0.11
LSA branching n (%)	2 (2.5%)	1 (6.7%)	1 (1.5%)	0.34
LSA plug occlusion, n (%)	13 (10.2%)	9 (18.8%)	4 (5.1%)	0.031

CAD: coronary artery disease, CSB: left carotid-subclavian bypass, LSA: left subclavian artery.

Supplementary Table S2

Covariate balance before and after propensity-score overlap weighting (threshold |SMD| < 0.10). Effective sample size after weighting: 67 no-CAD and 47 CAD patients.

Table S2. Covariate balance before vs. after overlap weighting.

Variable	SMD (unweighted)	SMD (weighted)
Propensity score	1.442	0.074
Age	0.874	0.000
Male sex	0.125	0.000
Hypertension	-0.179	-0.000
Diabetes mellitus	0.392	0.000
Hyperlipidemia	0.568	0.000
Chronic kidney disease	0.381	0.000
pAVD	0.631	0.000
cAVD	0.292	0.000
Current smoker	0.279	0.000

cAVD: cerebral arterial vascular disease, pAVD: peripheral arterial vascular diseases, SMD: standardized mean differences

Supplementary Figure S1

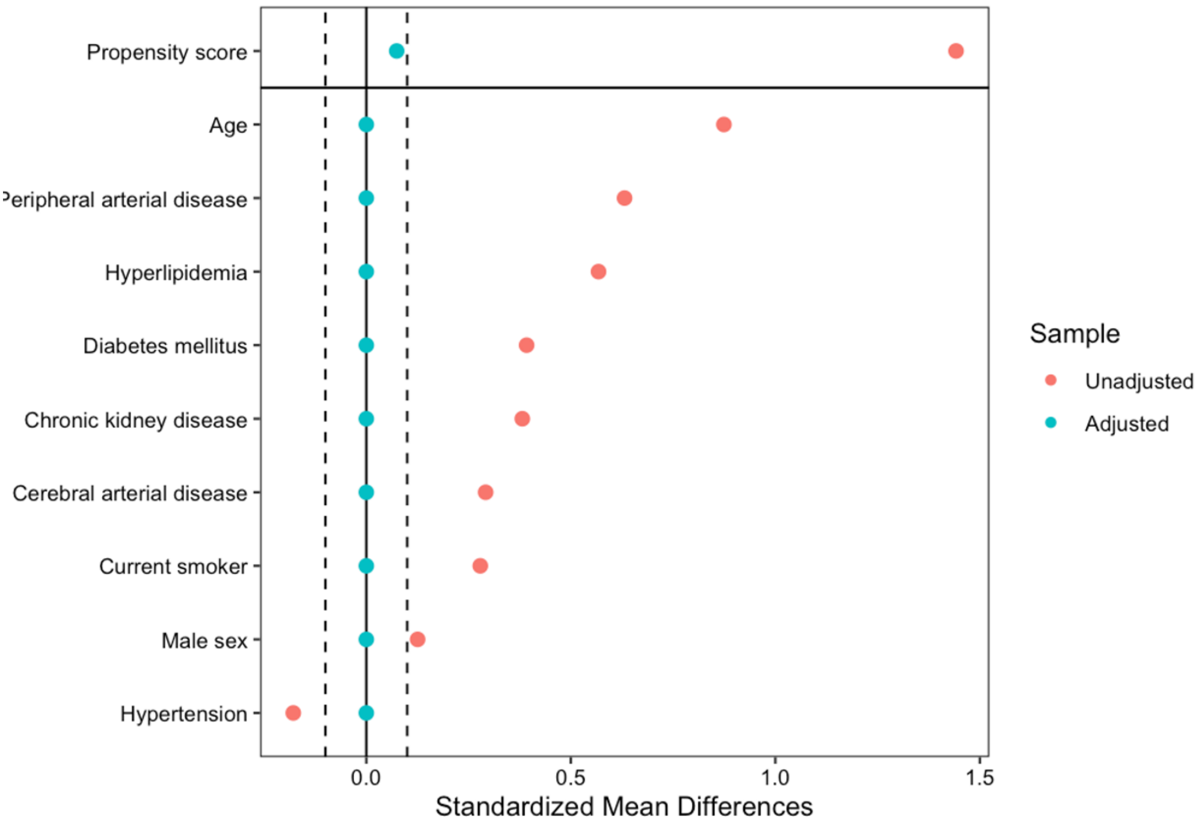

Figure S1. Love plot (overlap weighting). Covariate valance: unweighted vs. overlap weighted.

Supplementary Figure S2

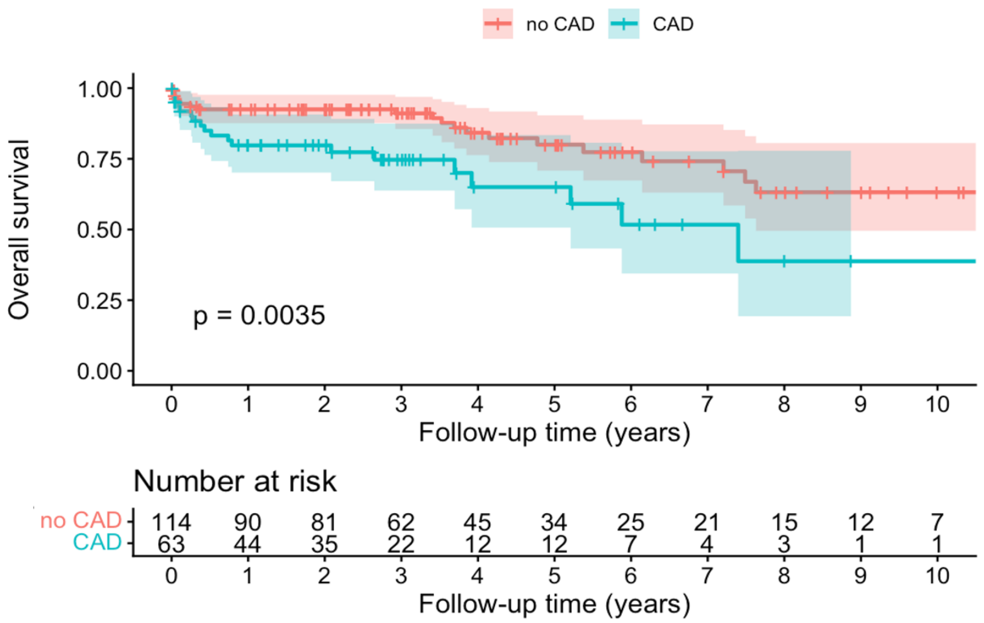

Figure S2. Kaplan Meier Curve for cumulative survival ratees of long-term mortality. CAD: coronary artery disease.
